# Innate immune responses in Behçet disease and relapsing polychondritis

**DOI:** 10.3389/fmed.2023.1055753

**Published:** 2023-06-26

**Authors:** Jun Shimizu, Masanori A. Murayama, Yoshihisa Mizukami, Nagisa Arimitsu, Kenji Takai, Yoshishige Miyabe

**Affiliations:** ^1^Department of Immunology and Parasitology, St. Marianna University of School of Medicine, Kawasaki, Kanagawa, Japan; ^2^Department of Animal Models for Human Diseases, Institute of Biomedical Science, Kansai Medical University, Hirakata, Osaka, Japan

**Keywords:** Behçet disease, relapsing polychondritis, neutrophils, monocytes, macrophages, cytokines, autoinflammatory disease, autoimmune disease

## Abstract

Behçet disease (BD) and relapsing polychondritis (RP) are chronic multisystem disorders characterized by recurrent flare-ups of tissue inflammation. Major clinical manifestations of BD are oral aphthae, genital aphthous ulcers, skin lesions, arthritis, and uveitis. Patients with BD may develop rare but serious neural, intestinal, and vascular complications, with high relapse rates. Meanwhile, RP is characterized by the inflammation of the cartilaginous tissues of the ears, nose, peripheral joints, and tracheobronchial tree. Additionally, it affects the proteoglycan-rich structures in the eyes, inner ear, heart, blood vessels, and kidneys. The mouth and genital ulcers with inflamed cartilage (MAGIC) syndrome is a common characteristic of BD and RP. The immunopathology of these two diseases may be closely related. It is established that the genetic predisposition to BD is related to the human leukocyte antigen (HLA)-B51 gene. Skin histopathology demonstrates the overactivation of innate immunity, such as neutrophilic dermatitis/panniculitis, in patients with BD. Monocytes and neutrophils frequently infiltrate cartilaginous tissues of patients with RP. Somatic mutations in UBA1, which encodes a ubiquitylation-related enzyme, cause vacuoles, E1 enzyme, X-linked, autoinflammatory, somatic syndrome (VEXAS) with severe systemic inflammation and activation of myeloid cells. VEXAS prompts auricular and/or nasal chondritis, with neutrophilic infiltration around the cartilage in 52–60% of patients. Thus, innate immune cells may play an important role in the initiation of inflammatory processes underlying both diseases. This review summarizes the recent advances in our understanding of the innate cell-mediated immunopathology of BD and RP, with a focus on the common and distinct features of these mechanisms.

## 1. Introduction

Behçet disease (BD) is an inflammatory disorder characterized by the frequent occurrence of oral ulcers, genital aphthous ulcers, and uveitis, with clinical manifestations involving the skin, cardiovascular, intestinal, and central nervous system (CNS) (1). These manifestations are important for diagnosis as there are no clinical or laboratory findings specific to BD. In 1985, an international study group developed diagnostic criteria based on the major symptoms of BD; a diagnosis is made when an individual has developed recurrent oral ulceration (at least three times over the past 12 months) with at least two of the following symptoms: persistent genital ulceration; eye lesions, such as uveitis and retinal vasculitis; skin involvement, such as erythema nodosum and thrombophlebitis; and a positive pathergy test (2). The clinical diagnostic criteria must be followed by the exclusion criteria for patients with other immune disorders presenting with common symptoms of BD. For example, chronic oral ulcerations are frequently observed in Crohn disease too (3). Additionally, Vogt-Koyanagi-Harada and Cogan syndromes should be considered during the differential diagnosis of BD in patients with uveitis (4, 5). CNS and gastrointestinal involvement are indicators of a poor prognosis in patients with neuro-BD and intestinal BD, respectively. Patients in the BD subgroup are difficult to distinguish from those with multiple sclerosis (6) and Crohn disease (7).

Relapsing polychondritis (RP) is a chronic inflammatory disorder characterized by chondritis of the auricular, nasal, joint, and tracheal cartilaginous tissues (8). In addition, it affects the proteoglycan-rich structures in the eyes, inner ear, heart, blood vessels, and kidneys. In the clinical features, respiratory involvement is associated with poor prognosis through the severe pulmonary complications such as tracheobronchomalacia and/or pulmonary infection (9). The diagnosis is usually made based on clinical symptoms using McAdam’s (10), Damiani’s (11), and/or Michet’s (12) criteria, because there are no pathognomonic clinical and laboratory features, similar to patients with BD. Approximately 20–35% of RP cases are further complicated by other immune disorders such as systemic vasculitis, rheumatoid arthritis, and systemic lupus erythematosus (10–13). In most instances, coexisting diseases precede the onset of RP (10), and in some patients, complications occur as consequent symptoms of RP (8). It is possible that the clinical course of the disease makes it difficult to obtain an early and accurate diagnosis.

**Figure 1:**
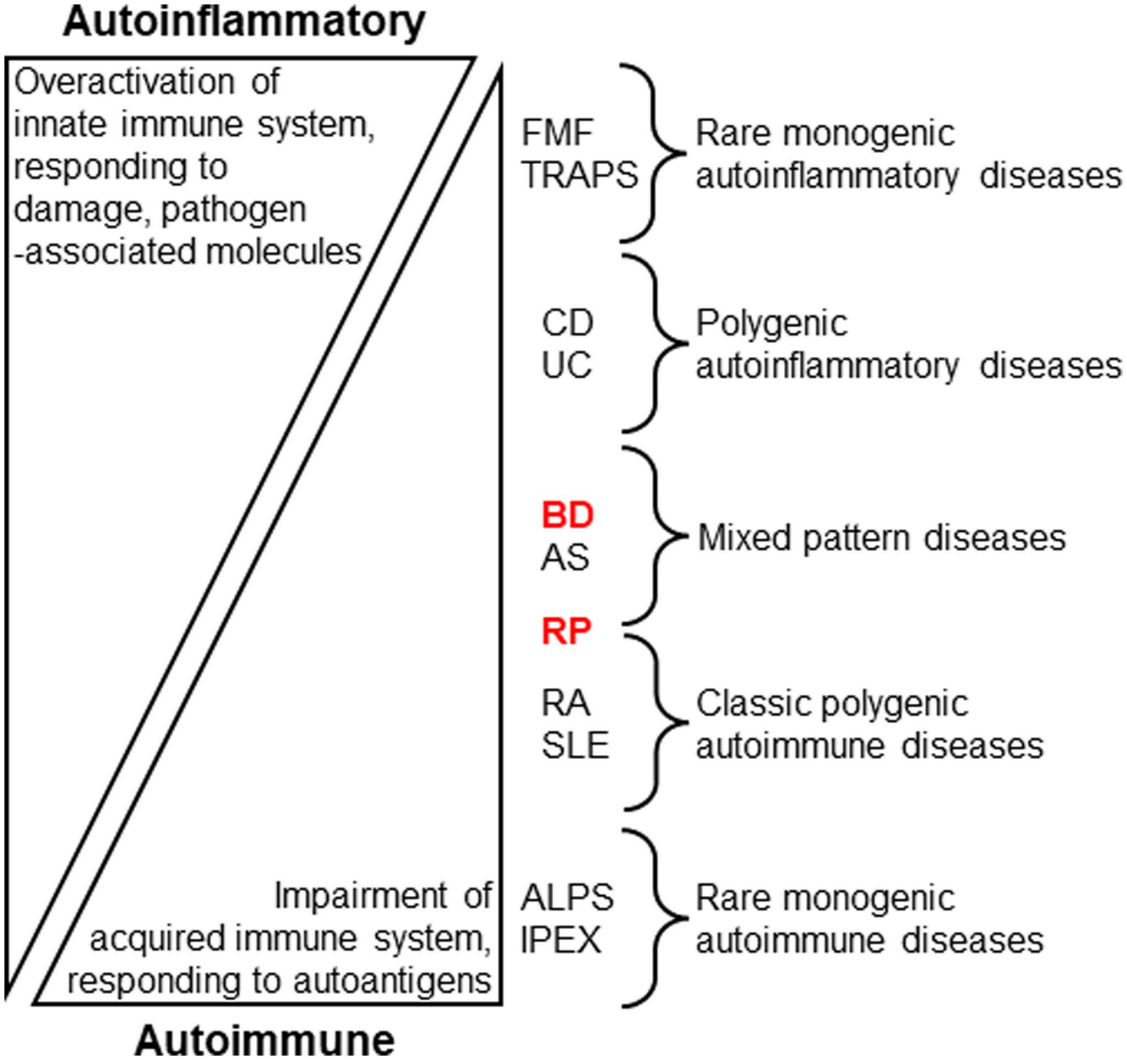
Stratification of human autoinflammatory and autoimmune diseases by evaluating immune conditions (14, McGonagle and McDermotte. PLoS Med. 2006; 3:e297. Modified). Innate immune overactivation via inflammatory cytokine signaling, pathogen sensing, and/or disruption of local tissue homeostasis are the proposed main causes of autoinflammatory diseases, while autoimmune diseases are associated with self-reactive lymphocytes through impaired immune tolerance. Behçet disease (BD) and relapsing polychondritis (RP) may be allocated to distinct clusters of the stratification. FMF: Familial Mediterranean fever; TRAPS: TNF receptor-associated periodic fever syndrome; CD: Crohn disease; UC: Ulcerative colitis; AS: Ankylosing spondylitis; RA: Rheumatoid arthritis; SLE: Systemic lupus erythematosus; ALPS: autoimmune lymphoproliferative syndrome; IPEX: immune dysregulation, polyendocrinopathy, enteropathy, X-linked.

Identifying new diagnostic biomarkers for human inflammatory diseases require further studies. Generally, human autoinflammatory diseases, such as familial Mediterranean fever (FMF) and tumor necrosis factor (TNF) receptor-associated periodic fever syndrome (TRAPS), are thought to be caused by abnormalities in phagocytes against pathogenic elements. In contrast, human autoimmune diseases are characterized by the overactivation of lymphocytes in response to autoantigens. In these studies, most immune disorders were suggested to be caused by a combination of autoimmune and autoinflammatory mechanisms in the disease spectrum, based on the genetic and cellular basis (14). In the immune disease spectrum, BD is associated with a mixed pattern of autoinflammatory and autoimmune diseases (Figure 1) (14).

In this review, we summarize current knowledge of innate cell mediated immunopathology in BD and RP to identify accurate positions of the immune disorders in the disease spectrum to facilitate the development of new therapeutic strategies.

## 2. Behçet disease

### 2.1. Epidemiology of BD

#### 2.1.1. Environmental factors in BD

BD is prevalent along the ancient Silk Road between the Mediterranean Basin and East Asia (1). The human leukocyte antigen (HLA)-B51 gene is established as a major BD susceptibility gene, especially in the patients with ocular involvement (1). Additionally, a geological association was observed between the prevalence of BD and HLA-B51 (15). These data demonstrated that BD inflammation may be triggered by innate immunity as well as environmental factors, such as bacterial and viral agents.

Oral plaque index scores are associated with the presence of oral ulcers and BD severity (16). Dental plaque bacteria (*Streptococcus sanguinis*) are frequently observed in the oral cavity of patients (17). Mouthwashes containing soluble betamethasone, doxycycline, and nystatin improve oral ulcer severity scores in patients with BD (18).

**Figure 2:**
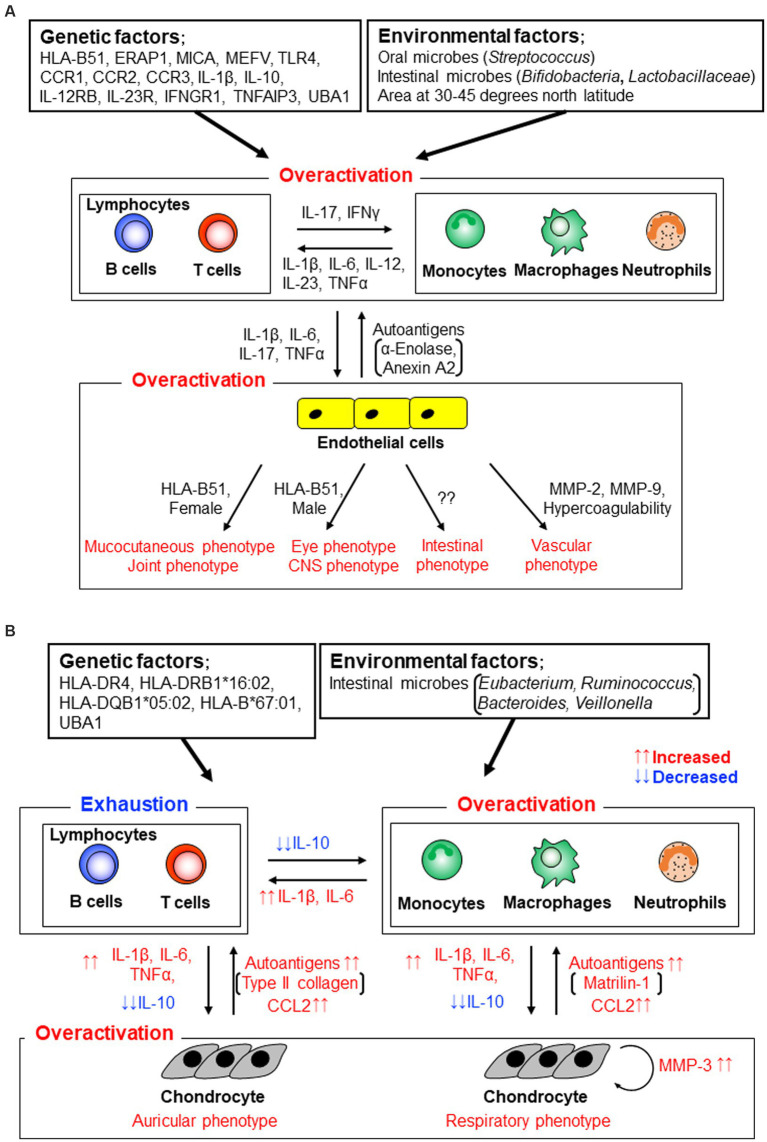
Stratification of human autoinflammatory and autoimmune diseases by evaluating immune conditions (14, McGonagle and McDermotte. PLoS Med. 2006; 3:e297. Modified).

Recent studies have revealed perturbation of oral and gut microbiota, especially increases in lactate-producing bacteria such as *Lactobacillus* and *Bifidobacterium*, in patients with BD compared with those in healthy individuals. Researchers have suggested pathological relationships between microbiota and immunological dysfunction in BD (Figure 2A) (19–22). In contrast to these clinical and laboratory findings, HLA-B51 transgenic mice demonstrate no obvious clinical phenotypes of BD, although stimulated neutrophils produce high levels of superoxide (23).

#### 2.1.2. Genetic variations in BD

Genome-wide profiling analyses revealed that, adding to HLA-B51, myeloid immune cell-related molecules, such as endoplasmic reticulum aminopeptidase-1 (ERAP1), major histocompatibility complex (MHC) class I polypeptide-related sequence-A (MICA), familial Mediterranean (MEFV) gene products, toll-like receptor-4 (TLR-4), c-c motif chemokine receptors CCR1-CCR3, interleukin (IL)-1β, IL-10, interferon (IFN)-γ receptor (IFNGR)-1, IL-23R, and IL-12RB, were risk factors of BD (24–29). These findings suggest that innate immune functions and bacterial identification systems play crucial roles in the pathogenesis of BD (Figure 2A). Lymphocytes obtained from BD patients react with human and/or mycobacterial heat shock protein peptides (30, 31). TLR-1, 2, and 4 are expressed more abundantly on neutrophils, monocytes, and lymphocytes derived from patients with BD than on those derived from healthy individuals (32).

The tumor necrosis factor-α-induced protein-3 (TNFAIP3) gene encodes A20 which regulates negatively TNFα pathway through the ubiquitin ligase activity (33). Patients with A20 haploinsufficiency develop BD phenotypes with an onset in childhood and young adulthood. Peripheral blood mononuclear cells (PBMCs) of the patients produced higher amounts of proinflammatory cytokines, such as IL-1β, TNFα, IL-17, and IL-18, in the presence of lipopolysaccharide (LPS), than those of healthy individuals.

#### 2.1.3. Clinical phenotypes in BD

Recent clustering analyses have demonstrated that patients with BD can be divided into several subgroups according to their clinical symptoms to simplify and increase the accuracy of the clinical assessment (34, 35). For example, patients with mucocutaneous manifestations belonged to one subgroup, those with vascular manifestations belonged to another, and those with eye and/or CNS involvement belonged to another (35).

Interestingly, HLA-B51 positivity was relatively low in patients with BD with intestinal involvement, and male and female patients had eye and mucocutaneous involvement, respectively (Figure 2A) (34).

### 2.2. Histopathology of BD

Erythema nodosum (EN)-like lesions and papulopustular lesions are common in patients with BD (36). Histopathological examination of EN-like lesions in BD demonstrates panniculitis with vasculitis, leukocytoclastic and lymphocytic vasculitis, or phlebitis of the dermis. BD skin lesions are occasionally associated with thrombosis. The infiltrating immune cells consist mainly of neutrophils and lymphocytes (37). Similarly, in papulopustular lesions, neutrophilic infiltration was observed in the epidermis and around hair follicles. Lymphocytic infiltration has been suggested to occur as a late-stage inflammation in the neutrophilic reaction (38). Indeed, an investigation of patients with EN-like lesions revealed that neutrophilic dermatitis/panniculitis was more frequently observed in patients with BD with EN-like lesions than in patients with nodular vasculitis or EN of other immune disorders (39).

Sterile needle pricks often form inflammatory papules or pustules on the skin in patients with BD, with infiltration of neutrophils and lymphocytes as a positive pathergy test (40). Higher response rates were observed for pricks with larger gauge and/or blunt needles in patients with BD (41). A similar procedure using saliva pricks increased positive rates and was associated with disease activity (42). These data suggest that pathergy tests with needle pricks lead to the overactivation of immune cells against pathogen- and damage-associated molecular patterns in patients with BD (40).

In the pathergy test, lymphocyte and monocyte infiltrations were persistent up to 48 h after the needle prick in patients with BD compared to healthy individuals (43). Small clusters of elastase-positive neutrophils have been observed at needle prick sites in the relatively early phases of the test until 24 h after the prick (44, 45).

In the oral and genital ulcer lesions, leukocytoclastic vasculitis and lymphocyte infiltration were frequently observed in the lamina propria of the lesions (46). Intestinal ulceration is commonly found in the ileocecal region and is histologically characterized by neutrophilic and lymphocytic cell infiltration around lesions (47–49). The postmortem brain tissues of patients with neuro-BD demonstrate perivascular cuffing of macrophages and T cells in the parenchyma (50).

### 2.3. Peripheral blood cells in BD

#### 2.3.1. Neutrophils in BD

Neutrophils produce reactive oxygen species (ROS) as a first-line defense against infectious pathogens (51). HLA-B51-positive neutrophils produce excessive superoxide compared to those without HLA in patients with BD and healthy individuals (23).

Neutrophil migration in patients with BD was enriched in an *in vivo* assay compared to that in healthy individuals, and the titers were significantly reduced in the disease remission phases (52). No significant differences were observed in superoxide production or adhesion capabilities between patients with BD and healthy individuals (52).

Neutrophil oxidative burst responses in patients with severe BD and organ involvement, such as complications of the eye, intestines, central nervous system, and cardiovascular system, were significantly higher than those in patients with mild BD (53).

#### 2.3.2. Neutrophil extracellular traps in BD

Neutrophils activated by phorbol myristate acetate, IL-8, or LPS become flat and produce extracellular structures called NETs, which contain myeloperoxidase, neutrophil elastase, and cathepsin G (54). The nuclear enzyme protein-arginine deiminase type 4 (PAD4) citrullinates histones and promotes chromatin decondensation (55). NETs degrade virulence factors and inhibit the growth of bacteria such as *Staphylococcus aureus*, *Salmonella typhimurium*, and *Shigella flexneri* (54). On the other hand, damage-associated molecular patterns, such as cholesterol crystals, induced NET release of neutrophils and the NETs with cholesterol crystals promoted IL-1β production of macrophages (56). PAD4 deficient mice demonstrated reduced NET formation and a lower degree of thrombosis (57).

NETs may increase and induce thrombosis in patients with BD. Neutrophil NET release and serum levels of DNA components were significantly increased in patients with active BD compared to those in patients with inactive BD and healthy individuals (58). Neutrophil PAD4 expression level is significantly higher in patients with BD than in healthy individuals (59). Thrombin generation parameters in platelet-poor plasma obtained from patients with BD were significantly higher than those in healthy individuals, and correlated well with DNA component levels (58). NETs obtained from BD patients effectively promoted IL-8 and TNFα production of monocytes/macrophages compared with healthy individuals (60). Diffuse elastase-producing neutrophils were observed in BD skin panniculitis and vasculitis (59). NETs play a crucial role in the pathogenesis of BD.

Low-density neutrophils are immature or degranulated and recognized in human diseases (61). The frequencies of low-density neutrophils and NET production by the stimulated cells were increased in patients with BD compared to healthy individuals, but the cells exhibited decreased phagocytic capacities (62). Determining the mechanisms underlying these associations warrant further studies.

#### 2.3.3. Monocytes/macrophages in BD

In *in vitro* experiments, bone marrow cells were differentiated into classically activated M1 macrophages in the presence of IFNγ and LPS and promoted production of proinflammatory cytokines, such as IL-1β, IL-6, and TNFα (63). Cells differentiate into alternatively activated M2 macrophages with IL-4 and increased IL-10 expression levels (63). M2 macrophages have been suggested to play distinct roles in lesions by reducing inflammation and promoting tissue remodeling.

Monocyte-derived macrophages treated with BD sera produced more effectively IL-12 and TNFα than those treated with sera of healthy individuals, suggesting M1 macrophage prevalence in peripheral blood of patients with BD (64). Stimulated M1 macrophages from patients with BD exhibit higher CCR1 expression levels than those from healthy individuals (65). Similarly, a gene expression profiling study demonstrated that, compared with healthy individuals, expression levels of proinflammatory monocyte-associated molecules, such as IL-1β and a CCR1 ligand CCL3, were elevated in patients with BD (66).

#### 2.3.4. Inflammasome components in BD

The inflammasome complex consists of a cytosolic nucleotide-binding domain, leucine-rich-repeat-containing (NLR) proteins, AIM2-like receptor (ALR) proteins, adaptor apoptosis-associated speck-like protein containing a CARD (ASC), and pro-caspase-1 (67). The well-studied inflammasome NLRP3 responds to and is activated by bacterial, fungal, and viral pathogen-associated molecular patterns and damage-associated molecular patterns such as ATP and uric acid crystals (68). Activated caspase-1 processes pro-IL-1β and pro-IL-18 and biologically active cytokines are secreted (68). An autoinflammatory disease, cryopyrin-associated periodic syndrome, has been suggested to be associated with NLRP3 gene mutations (69).

In the PBMCs of patients with BD, the protein and mRNA levels of NLRP3, ASC, and caspase-1 were significantly increased compared with healthy individuals (70, 71). Activated PBMCs with LPS and ATP induced significantly higher levels of IL-1β compared with cells without the stimulation (70). NLRP3 levels of cerebrospinal fluids of BD patients with CNS involvement are positively correlated with serum C-reactive protein concentrations and erythrocyte sedimentation rates (71). Patients with BD share common clinical features, at least among those with autoinflammatory diseases.

#### 2.3.5. Eosinophils in BD

Serum immunoglobulin E (IgE) and eosinophil counts are significantly reduced in patients with BD (72). Similarly, serum eosinophil cationic protein levels are significantly lower in active patients with BD than in inactive patients (73), suggesting a role for helper type 1 (Th1)-skewed cytokine responses in the pathogenesis of BD.

### 2.4. Humoral mediators in BD

#### 2.4.1. Cytokines/chemokines in BD

A literature-based meta-analysis ascertained that serum IL-1β, IL-6, and TNFα were significantly increased in patients with BD compared with healthy individuals (Figure 2A) (74). High levels of Th1 and Th17 related cytokines, such as IL-1β, IL-6, IL-12, IL-17, IL-23, IFNγ, and TNFα, were identified in an array analysis (75). BD shares skewed IL-17/IL-23 pathways and several clinical features with spondyloarthritis and Vogt-Koyanagi-Harada disease (76, 77). Serum and plasma levels of CCL2, CCL3, and CXCL10 are higher in patients with BD compared with healthy individuals (78–80). Aqueous humor CXCL16 and CX3CL1 levels are higher in patients with BD than in healthy individuals and patients with Vogt-Koyanagi-Harada disease, suggesting an enhancement of Th1 responses in BD uveitis (81).

#### 2.4.2. Matrix metalloproteinases in BD

Serum MMP-2 and MMP-9 levels were significantly higher in patients with vasculo-BD than in healthy individuals (Figure 2A) (82), especially in patients with aneurysms, similar to patients with abdominal aortic aneurysms (83). Synovial fluid concentrations of MMP-3 are significantly lower in patients with BD than in patients with rheumatoid arthritis and are comparable to those in patients with osteoarthritis (84).

#### 2.4.3. Autoantibodies in BD

Autoantibodies were observed in patients with BD and reacted with an endothelial cell antigen, α-enolase (a positive rate of 38% by an ELISA) (85), a ubiquitously expressed membrane protein, prohibitin (28% by an ELISA) (86), α-tropomyosin (22% by an ELISA) (87), the nuclear mitotic apparatus protein (NuMA; 28% by an ELISA) (88), a riboflavin-containing flavoprotein (41% by an ELISA) (89), a membrane protein annexin A2 (34% by an ELISA) (90), a microtubule-related protein, kinectin (23% by an immunoprecipitation assay) (91), and an actin-binding protein, cofilin-1 (13% by western blotting; Figure 2A) (92). Thus, BD demonstrates a mixed pattern of autoinflammatory and autoimmune diseases within the spectrum (14).

## 3. Relapsing polychondritis

### 3.1. Epidemiology of RP

#### 3.1.1. Genetic variations in RP

Epidemiological studies on patients with RP have identified that the incidence rates are approximately the same in several regions of the world (93–95). HLA-DR4 appears to be a susceptibility allele for RP (96). A recent genetic study demonstrated that HLA-DRB1*16:02, HLA-DQB1*05:02, and HLA-B*67:01 are associated with RP (Figure 2B) (97). Based on our data, the authors concluded that RP may be a distinct disease from other rheumatic diseases such as rheumatoid arthritis, systemic lupus erythematosus, Takayasu arthritis, and BD.

#### 3.1.2. Environmental factors in RP

Based on the global incidence rates mentioned above, few environmental factors have been reported to be associated with RP pathogenesis. Interestingly, similar to the data of patients with BD, a metagenomic analysis demonstrated characteristic alterations in the gut microbiota composition, such as an increase in the abundance of *Eubacterium*, *Ruminococcus*, *Bacteroides*, and *Veillonella*, in patients with RP compared with that in healthy individuals. Here, we suggest an association between gut microbes and RP immunopathogenesis (98).

#### 3.1.3. Clinical phenotypes of RP

In the clinical manifestations, respiratory and auricular involvement, which are two key hallmark features of RP, are recognized in 40–67% and 85–90% of patients with RP, respectively, at the latest follow-up (10–13). Tracheobronchial chondritis is increasingly recognized as distinct from other pathogenic complications (Figure 2B) (99, 100). Certainly, patients with RP with respiratory involvement have progressive disease compared to those with auricular involvement (101). Similar to the ocular involvement in BD, posterior segment inflammation is associated with a weak response to treatment (102).

### 3.2. Histopathology of RP

In the initial stages of the disease, mononuclear cells and neutrophils infiltrate the perichondrium beside the normal cartilage tissue (103, 104). Among the inflammatory cells in granulation tissues, CD4+ Th cells and CD68+ monocytes/macrophages are prevalent (105). Damaged chondrocytes produce MMP-3 and cathepsins, and the number of proteolytic enzyme-expressing cells correlates with that of apoptotic chondrocytes. Interestingly, MMP-3 was observed in the cartilage and perichondrium, whereas MMP-8 and MMP-9 were detected only in perichondrium granulation tissues. Cartilage tissues are progressively destroyed and finally replaced by fibrous connective tissues.

Notably, 1.6–38% of patients with RP showed skin involvement (9, 10, 12, 13, 95, 106); mucosal aphthosis, nodules on the limbs, purpura, and sterile pustules were the most common dermatological manifestations (107). Skin biopsy specimens revealed leukocytoclastic vasculitis, thrombosis of the skin vessels, septal panniculitis, neutrophil infiltration, and lymphocytic vasculitis as their histological findings. About 0–12% of patients with RP develop neurological manifestations, mainly confusion, seizures, delusions, amnesia, and/or dementia (108). Histopathology of the CNS exhibited perivascular cuffs of monocytes/lymphocytes and lymphocytic infiltration in the meninges and the cerebral parenchyma of patients with RP (109–111). In contrast to BD, gastrointestinal involvement is not generally identified in patients with RP (8–13, 93, 95, 106).

### 3.3. Peripheral blood cells in RP

#### 3.3.1. Neutrophils in RP

As mentioned previously, neutrophil infiltration into cartilage tissues has been recognized since the early stages of chondritis (103, 104). Leukocyte clastic vasculitis and neutrophil infiltration are frequently observed (40%) in skin biopsy specimens (107). These results suggested that neutrophil activation plays a crucial role in the initiation of chondritis in patients with RP.

#### 3.3.2. Monocytes/macrophages in RP

Gene expression level of IL-10, a major effector cytokine of regulatory T (Treg) cells, was significantly higher in freshly isolated PBMCs from patients with RP than in those from healthy individuals (112). After the initiation of cell culture with mitogen stimulation, IL-10 gene expression level was significantly decreased in patients with RP compared to that in healthy individuals. The researchers suggested that the gene expression analysis of PBMCs revealed Treg cell exhaustion or anergy of patients with RP and the skewed T cell function associated with innate cell overactivation (Figure 2B) (113).

#### 3.3.3. Eosinophils in RP

Similar to neutrophil infiltration in RP lesions, eosinophils have been identified in specimens from the conjunctiva (114), nasal septum (104), and skin (115), probably indicating their early involvement in the process of chondritis in patients with RP.

### 3.4. Humoral mediators in RP

#### 3.4.1. Cytokines/chemokines in RP

In Th cell-related cytokines, IFNγ and IL-10 were increased in the sera of patients with RP compared with healthy individuals (116, 117). Serum levels of innate cytokines and chemokines, such as IL-8, CCL2, and CCL4, were higher in patients with RP than in healthy individuals (Figure 2B) (116, 117).

#### 3.4.2. MMPs in RP

Serum MMP-3 levels were higher in patients with RP than in healthy individuals, likely corresponding to histopathological changes in the patients (101, 116). Lymphocytes, monocytes/macrophages, and MMP-3 positive chondrocytes were simultaneously observed in RP lesions, suggesting that these cells aggravate chondritis (105). When the concentrations were compared between RP patients with and without respiratory involvement based on the epidemiological data mentioned above, MMP-3 levels increased significantly in patients with respiratory involvement compared to those without respiratory involvement (118).

In an *in vitro* assay, RP PBMCs upregulated mRNA expression of inflammatory cytokines IL-1β and IL-6 against stimulation compared with those of healthy individuals (118). Expression positively correlated with serum MMP-3 only in patients with RP and respiratory involvement. These data suggested that mononuclear cells with innate cytokines play a crucial role in the inflammatory processes of RP lesions, especially in patients with RP and respiratory involvement (Figure 2B).

#### 3.4.3. Triggering receptor expressed on myeloid cell in RP

As the molecule name, neutrophils and monocytes/macrophages express TREM-1 and promote inflammation partly through TLR-4 pathway activation (119). Soluble TREM-1 is increased in the sera of RP patients with active disease compared with those with inactive disease, suggesting its possible role as a biomarker (116).

#### 3.4.4. Autoantibodies in RP

Several cartilage elements were identified as potential autoantigens for RP (Figure 2B). An initial report of circulating autoantibodies in patients with RP revealed that, using indirect immunofluorescence, 33% of patients had autoantibodies against type 2 collagen, and the titers increased when acute symptoms were exhibited (120). Type 2 collagen-immunized rats develop auricular chondritis in the presence of type 2 collagen-reactive antibodies (121).

Matrilin-1 is a cartilage-specific protein, and its serum concentrations were found to be significantly elevated in an RP patient with tracheal chondritis who was monitored for 2 years (122). Autoantibodies for matrilin-1 were detected using ELISA in 13% of 97 patients with RP (123). In this study, researchers ascertained that sera from RP patients with positive anti-matrilin-1 antibodies reacted with newborn mouse tracheolaryngeal cartilage, whereas sera from patients with rheumatoid arthritis did not.

Certainly, several cartilage components are associated with phenotypic differences between patients with RP with and without respiratory involvement.

## 4. VEXAS and RP

A cutting-edge analysis demonstrated that patients with somatic mutations in UBA1, a gene encoding the ubiquitin activating enzyme E1, developed treatment-refractory severe autoinflammatory conditions in late middle age, such as vacuole, E1 enzyme, X-linked, autoinflammatory, and somatic syndrome (VEXAS) (124). It is characterized by refractory constitutional symptoms, ear and nose chondritis, and inflammatory arthritis. Patients with VEXAS often develop hematological disorders, such as myelodysplastic syndrome (MDS) and multiple myeloma, with a poor prognosis. Hypercellular bone marrow, vacuolization of erythroid and myeloid precursors, and spontaneously activated peripheral blood myeloid cells are common laboratory findings in patients with VEXAS.

When the symptoms were compared between RP patients with and without VEXAS, fever, ear chondritis, skin involvement (leukocytoclastic vasculitis and neutrophilic dermatosis), and periorbital edema were frequently observed in the patients with the syndrome (125). Notably, RP patients with VEXAS do not develop tracheobronchial chondritis during their clinical course. These data support the hypothesis that local interactions between inflammatory myeloid cells and chondrocytes/extracellular matrix are important for the initiation of chondritis in patients with RP.

## 5. Myelodysplastic syndrome in BD

A recent case report demonstrated that a 60-year-old man with somatic variants of UBA1 developed BD phenotypes with MDS and was resistant to aggressive treatments (126). This report demonstrates the possibility that the clinical spectrum of VEXAS can expand to BD manifestations.

In epidemiological studies, 10–20% of patients with MDS developed autoimmune manifestations (127, 128); conversely, autoimmune manifestations proceeded with the onset of MDS in 30% of patients (129). The prevalent autoimmune manifestations in a retrospective cohort study were neutrophilic dermatoses, such as Sweet syndrome, pyoderma gangrenosum, and BD (127). In this study, the deletion of 5q and trisomy 8 were associated with neutrophilic dermatosis and BD, respectively.

## 6. Mouth and genital ulcers with inflamed cartilage syndrome

Patients with MAGIC syndrome exhibit clinical features of both BD and RP. A prospective cohort study demonstrated good sensitivity in classifying patients according to McAdam’s or Damiani’s Criteria for RP and the International Criteria for BD (130). Interestingly, in this study, RP patients with MAGIC syndrome demonstrated higher frequency of anti-type 2 collagen autoantibodies than in those without MAGIC syndrome. This finding suggests differences in the underlying molecular mechanisms between the two respective groups of patients.

## 7. Innate immune responses in treatment of BD and RP

Tables 1, 2 show previously reported data on innate immune responses to therapeutic treatment in BD and RP, respectively (59, 131–149).

**Table 1:** Innate immune responses *in vivo* and *in vitro* induced by immunosuppressants in patients with Behçet disease.

References	Patient numbers	Medication	Study protocols	Laboratory findings
(131)	80	Colchicine	Peripheral blood samples were obtained before and 1, 3 months after the initiation	Neutrophil-lymphocyte and monocyte-lymphocyte ratios decreases
(132)	61	Colchicine, methyl-PSL, PSL	Peripheral blood samples were obtained from active patients with and without the treatment before and 1, 3 months after the initiation	Neutrophil-lymphocyte ratio decrease, plasma IFNγ, IL-4 decease
(59)	31	Colchicine, dexamethasone, Cl-amidine, N-Acetyl cysteine	Neutrophils were obtained and incubated with compounds (*ex vivo*)	NETS release decrease (all compounds)
(133)	10	Colchicine	Neutrophils and monocytes were obtained and incubated with colchicine (*ex vivo*)	Oxidative burst decrease, ROS production decrease
(134)	N/A	Colchicine	Neutrophils were obtained and incubated with colchicine (*in vitro*)	NET release decrease, intracellular ROS levels not change
(135)	35	Colchicine, PSL	Peripheral blood samples were obtained from active patients with and without treatment	Neutrophil CXCR2 expression decrease only by corticosteroid
(136)	8	Infliximab	Peripheral blood samples were obtained before and 1 week after infliximab infusion	IFNγ, IL-6, TNFα production not change in active patients
(137)	5	Infliximab	CSF and sera were obtained before, 1 day, and 3, 7, 18 weeks after infliximab infusion	CSF IL-6 decrease, CSF TNFα and serum IL-6 not change
(138)	18	Infliximab	Peripheral blood samples were obtained before and 1 day after infliximab infusion (*ex vivo*)	TNFα decease and IFNγ, IL-12R increase
(139)	7	Gevokizumab	Peripheral blood samples were obtained before and 7 days after infliximab infusion (*ex vivo*)	IL-1β decrease and IL-1ra not change
(140)	12	Colchicine, apremilast	Neutrophils were collected before and 12 weeks after treatment and incubated with colchicine and apremilast (*ex vivo*)	NET release and CD11b, CD64, CD66b-expressing cells decrease by both
(141)	10	Azithromycin	Peripheral blood samples were obtained and incubated with azithromycin (*ex vivo*)	IFNγ production decrease
(142)	50	Zinc supplementation	Patients were randomly allocated into zinc gluconate or placebo groups for 12 weeks	Caspase-1/NLRP3 expressions, and serum IL-1β decrease

PSL, prednisolone. Cl-amidine, an inhibitor for PAD4 (a key enzyme of NET formation). N-Acetyl cysteine, a reactive oxygen species (ROS) inhibitor. N/A: not applicable. Infliximab: anti-TNF antibody, Gevokizumab: anti-IL-1β antibody.

**Table 2:** Induction of innate immune responses by administration of immunosuppressants in patients with relapsing polychondritis.

References	Age/gender	Clinical phenotypes	Medications	Previous medications	Clinical responses	Laboratory findings
(143)	29/f	Respiratory	Tocilizumab	PSL, CsA, TAC, CP, infliximab	PSL reduction to 10 mg/day	Serum MMP3 decrease
(143)	52/m	Respiratory	Tocilizumab	PSL, MTX	PSL reduction to 10 mg/day	Serum MMP3 decrease
(144)	65/f	Respiratory	Tocilizumab	PSL, CP	PSL reduction to 0 mg/day	Serum IL-6 decrease
(145)	55/m	Respiratory	Adalimumab	PSL, MTX, AZA	PSL reduction to 8 mg/day	Serum MMP3 decrease
(146)	68/m	Auricular	Adalimumab	PSL	PSL reduction to 20 mg/day	Serum IL-6, Anti-type 2 collagen antibody titer decrease
(147)	18/m	Respiratory	Infliximab, Etanercept	PSL, MTX, CsA	MTX resumption after biologic therapy cessation	Serum IFNγ, IL-2, IL-12 decrease
(148)	61/m	Auricular + meningoencephalitis	Methyl-PSL	None	Follow-up with PSL and MTX	CSF IL-6 decrease
(149)	66/m	VEXAS	Tocilizumab	PSL	PSL dose before 9 mg/day to 3 mg/day for 8 months	High levels of IL-6, IL-1β, IFNγ, TNFα sustained
(149)	74/m	VEXAS	Tocilizumab	PSL, MTX	PSL dose before 22.5 mg/day to 13.5 mg/day for 5 months	High levels of IL-6, IL-1β, IFNγ sustained
(149)	67/m	VEXAS	Tocilizumab	PSL, AZA, colchicine	PSL dose before 30 mg/day to 30 mg/day for 5 months	High levels of IL-6, IL-1β, IFNγ, TNFα sustained

PSL, prednisolone; CsA, cyclosporine; TAC, tacrolimus; CP, cyclophosphamide; MMP, matrix metalloproteinase; MTX, methotrexate; AZA, azathioprine; CSF, cerebrospinal fluid; VEXAS, vacuole, E1 enzyme, X-linked, autoinflammatory, somatic syndrome. Tocilizumab: anti-IL-6R antibody, Adalimumab: anti-TNF antibody, Infliximab: anti-TNF antibody, Etanercept: anti-TNF antibody.

Colchicine reduced neutrophil and monocyte infiltration into lesions by decreasing the expression levels of adhesion molecules, such as selectin P ligand (SELPLG) and platelet endothelial cell adhesion molecule-1 (PECAM-1) (150). In a study using BD neutrophils, colchicine reduced NET release to an extent similar to that of methylprednisolone, a PAD inhibitor (Cl-amidine), and an ROS inhibitor (N-acetyl cysteine) (59). Biological agents are recommended for the treatment of patients with refractory BD and RP (76, 151).

A phosphodiesterase (PDE)-4 inhibitor, apremilast, reduces degradation of cyclic adenosine monophosphate and inhibits production of proinflammatory cytokines such as IL-12, IL-23, and TNFα from PBMCs (152). Additionally, apremilast decreased the total number of oral ulcers during a 12-week placebo-controlled clinical trial (153).

Zinc plays a crucial role in innate and adaptive immune function and its depletion has led to IL-1β secretion increase of stimulated macrophages through induction of NLRP3 inflammasome (154). Zinc gluconate supplementation reduced the expression levels of serum IL-1β and white blood cell NLRP3, while decreasing the incidence of genital ulcer in patients with BD within 3 months (142).

Sustained high concentrations of inflammatory cytokines have been observed in patients with VEXAS, indicating the refractory nature of the disease, even after the initiation of an anti-IL-6 agent, tocilizumab, administration (149).

## 8. Conclusion

This review collates and summarizes the recent advances in our understanding of the innate cell mediated immunopathology of BD and RP. These studies suggest that innate immune cells are crucial in the direct initiation of local inflammation under dysregulated lymphocyte function in inflammatory diseases (Figure 1). Certain susceptibility genes characterizing BD are associated with several rare monogenic autoinflammatory diseases, such as FMF and TRAPS. Meanwhile, RP is characterized by distinct clinical phenotypes that are associated with several autoantigens in humans and mice. Adaptive immune cells and genetic/environmental factors simultaneously enhance innate immune responses in BD and RP. The identification of controllable active elements is important for the development of effective and safe treatment approaches.

## Author contributions

JS and MM conceived and prepared the manuscript. YMiz, NA, KT, and YMiy prepared and edited the manuscript. JS, MM, YMiz, NA, KT, and YMiy approved the published version of the manuscript.

## Funding

The work of YMiy is supported by the Japanese Society for the Promotion of Science (JSPS) KAKENHI grant number JP22K08531 and AMED under Grant Number 22jm0210069h004 and 22jm0610070h0001, Kato Memorial Bioscience Foundation, The Naito Foundation and The Uehara Memorial Foundation, MM is supported by JSPS KAKENHI (JP21K06955 and JP21H02394), SRF foundation (2022Y003), Kansai Medical University alumni association (Katano Prize) and Kansai Medical University Molecular Imaging Center of Diseases. The work of JS and MM is supported by JSPS KAKENHI (JP22K08532 and JP23K05618). The work of NA and MM is supported by JSPS KAKENHI (JP21K07379).

## Conflict of interest

The authors declare that the research was conducted in the absence of any commercial or financial relationships that could be construed as a potential conflict of interest.

## Publisher’s note

All claims expressed in this article are solely those of the authors and do not necessarily represent those of their affiliated organizations, or those of the publisher, the editors and the reviewers. Any product that may be evaluated in this article, or claim that may be made by its manufacturer, is not guaranteed or endorsed by the publisher.
